# A Sketch of an Epidemic of Dengue or Breakbone Fever, as It Prevailed in Wilmington, N. C., in the Autumn of the Year 1860

**Published:** 1861-03

**Authors:** James H. Dickson


### *A Sketch of an Epidemic of Dengue or Breakbone Fever, as it Prevailed in Wilmington, N. C., in the Autumn of the Year*, 1860. By James H. Dickson, M. D.

Synonyms, *Dingee, Dunga, Dandy, Bouquet, Breakbone Fever.*

In the foregoing barbarous nomenclature, the curious philologist will be apt to find his ingenuity puzzled and his industry baffled, in his attempt to trace the origin of a word which has been adopted to designate a very marked and peculiar form of disease, without being furnished with that "*filum labarynthi*," which its history affords.

It certainly furnishes a rare instance of a word finding its way into scientific treaties from so humble an origin; for it carries with it the evidence of its negro parentage, not patent enough to be discovered by any ordinary process of word-hunting, and yet sufficiently obvious when the inquirer has been started on the proper track.

The West Indian negro gave it the name of Dandy fever, from the stiff gait which it caused its subjects to assume, when suddenly seized with it, as they sometimes were, when passing along the street. When it appeared in Cuba, this name, in undergoing the Spanish pronunciation, was changed into Dunga or Dengue, which it still retains.

This very unique and peculiar affection, has never, so far as I have been able to learn, prevailed in this place before the occurrence of the epidemic of which I shall now endeavor to give some account.

It seems indeed to be an unfrequent visitor of any locality, rarely, if ever appearing, for two consecutive seasons in any place.

The first notice which we have of this form of fever, is in a paper by Dr. Jas. Mellis, giving an account of its prevalence in Calcutta, in the year 1824.

In the latter part of 1827, it made its appearance in the West India islands. In the Spring of 1828, it broke out in New Orleans, and in the fall of the same year it prevailed in Charleston and Savannah. Papers descriptive of the disease as it prevailed in the latter cities, have been published by S. H. Dickson and W. R. Waring.

It would thus seem to be a tropical or tropicoid disease, and yet if we can rely upon the correctness of the opinion which regards the *so-called* breakbone fever, which Dr. Rush describes as prevailing in the autumn of 1780, in the city of Philadelphia, as the disease in question, or the true Dengue, it would seem occasionally to transcend its native limits.

Dr. Rush describes the Philadelphia epidemic as a bilious *remittent* fever to which the name of breakbone fever was popularly applied in consequence of the severity of the pains with which it was attended.

Not having at hand Dr. Rush's description of the disease, I am in some doubt whether to regard that instance as one of the genuine Dengue, or only as a form of remittent fever, with the usual spinal and neuralgic pains more than usually developed.

Such varieties of remittent fever are not very rare, but they must not be confounded with this very peculiar affection.

The genuine Dengue seems to be a complex malady, combining some or all of the characteristic features of our ordinary autumnal remittent fever, with very marked symptoms of rheumatism, together with, in many cases, an eruption, closely resembling in some instances urticaria, and in others scarlatinous erythema. Occasionally, the eruption assumed a miliary, and in some instances a rubeolous appearance.

In those instances in which the eruption did not show itself during the progress of the case, the occurrence of cutaneous desquamation at the close of the attack, left no doubt as to its true character.

This form of fever seems to exhibit no very marked prodromata. In some rare instances it makes its approach insidiously, but for the most part it attacks with abrupt suddenness.

A slight chilliness ordinarily ushers in a febrile paroxysm of greater or less severity, accompanied with head ache, chiefly in the back of the head, and distressing pains in the loins and in the course of the large nerves in the lower extremities.

The entire cerebro-spinal nervous system, as well as the peripheral extremities of the nerves, as in the great centres gave token of its pathological condition.

In some instances, the chill was of very marked intensity, and the febrile reaction in such cases was apt to be of corresponding severity.

Restlessness and a sense of great debility are apt to be prominent attendant symptoms.

The pains in the limbs and joints described as rheumatic, though for the most part uniform in their resemblance to that disease, were occasionally seemingly capricious in their character, attacking the smaller joints, and now and then, the stomach and bowels, giving the case in these features no slight resemblance to gout.

In our epidemic, an attack of Dengue was by no means uniform in its duration or its intensity. Indeed it ran through every gradation from the mild ephemeron, scarcely requiring any treatment, to a grave form of fever of ten or twelve days' duration.

A striking feature in most cases of any severity, was nausea and precordial distress. In some instances the gastric irritability was a very prominent symptom, and one which, by its obstinate persistence, seriously embarrassed the treatment.

It is usual to ascribe the occurrence of this disease to epidemic influence, and in the present state of our knowledge, it is perhaps impossible to assign it a more specific origin.

This epidemic influence or occult cause would seem to have engrafted itself (in our epidemics) on the ordinary morbific cause of autumnal remittent fever, for the cases evidently partook largely of that character, though in some of its features it was almost Protean, for we might, with strict propriety, characterise one case as rheumatic, another as gout, another as scarlatinous, &c.

It seemed to be no respecter of age or sex, all classes being equally liable to be attacked by it.

A very small fatality seems to be a notable characteristic of the Dengue; for though many suffered severely and some were seriously threatened, I believe no fatal case occurred.

Some of the writers who have given us a description of this form of fever, think that they have discovered in it a resemblance to the yellow fever, in the circumstance of its having but one paroxysm, with a long and delusive intermission, followed in the one case by a return of a fatal exacerbation, and in the other by a febrile paroxysm, which undergoes solution by the occurrence of a cutaneous exanthem.

I am constrained, however, to regard this as a forced and painful analogy. At any rate, such a form of the disease was not observed in the epidemic which I am endeavoring to sketch in this paper.

Instead of the peculiar paroxysmal form of yellow fever, this epidemic assumed the garb of *bilious remittent*, with daily exacerbations and remissions.

Its want of fatality, too, is a feature which separates it widely from yellow fever, and the extinction by the occurrence of frost, is equally characteristic of bilious and yellow fever.

The most probable conclusion from all our observations and comparisons, would seem to be, that the Dengue is Protean in its phases, and "*sui generis*" in its nature.

*Sequelæ*—A very striking and somewhat protracted debility was a not unfrequent sequel of the disease.

In some instances, among children, a paralysis in the lower extremities occurred, and was of conisderable duration; but I believe this serious sequel of the malady has not been permanent in any instance. Dr. W. G. Thomas informs me that several cases of this kind fell under his observation, all of which have recovered or are convalescing.

The occurrence of such a sequel of the disease as this, indicates that the nervous centres bear very largely the onus of the disease, and exhibits an intensity of action of the morbific cause, far exceeding the action of the cause of most other forms of fever upon the spinal axis.

When paralysis occurs as a result of convulsive-remittent fever in children, it almost invariably assumes the form of hemiplegia, and is generally of very transient duration.

*Treatment.*—Many of the milder cases were treated by the exhibition of a mild aperient and a sudorific anodyne. The severer cases seemed to call for the treatment which has been found needful and appropriate in our ordinary form of bilious fever. A mercurial cathartic (generally a pill of mass. hydrarg.) followed by a saline aperient, or a dose of calcined magnesia, was administered at the invasion of the attack. This was followed by the use of sulph. quinine, with or without the addition of an opiate as circumstances seemed to indicate.

In the large majority of cases, the opiate was found indispensable, for in by far the larger proportion of the cases, the spinal and articular pains were decidedly pronounced and called loudly for their administration.

Diffusible stimuli were found useful in some cases, in which unusual debility and prostration occurred; but the great mass of the cases were conducted to a satisfactory convalescence by the use of mild tonics.—*Medical Journal of North Carolina.*

